# The Genome of “*Candidatus* Liberibacter asiaticus” Is Highly Transcribed When Infecting the Gut of *Diaphorina citri*

**DOI:** 10.3389/fmicb.2021.687725

**Published:** 2021-07-12

**Authors:** Josiane Cecília Darolt, Flavia de Moura Manoel Bento, Bruna Laís Merlin, Leandro Peña, Fernando Luis Cônsoli, Nelson Arno Wulff

**Affiliations:** ^1^Instituto de Química, Universidade Estadual Paulista “Julio de Mesquita Filho” – UNESP, Araraquara, Brazil; ^2^Departamento de Pesquisa & Desenvolvimento, Fundo de Defesa da Citricultura – Fundecitrus, Araraquara, Brazil; ^3^Laboratório de Interações em Insetos, Departamento de Entomologia e Acarologia, Escola Superior de Agricultura Luiz de Queiroz, Universidade de São Paulo, Piracicaba, Brazil; ^4^Instituto de Biologia Molecular y Celular de Plantas – Consejo Superior de Investigaciones Científicas, Universidade Politécnica de Valencia, Valencia, Spain

**Keywords:** greening, HLB, prophage, psyllid, Las, ACP, metatranscriptomics

## Abstract

The Asian citrus psyllid, *Diaphorina citri*, is the vector of the bacterium “*Candidatus* Liberibacter asiaticus” (Las), associated with the devastating, worldwide citrus disease huanglongbing. In order to explore the molecular interactions of this bacterium with *D. citri* during the vector acquisition process, cDNA libraries were sequenced on an Illumina platform, obtained from the gut of adult psyllids confined in healthy (H) and in Las-infected young shoots (Las) for different periods of times (I = 1/2 days, II = 3/4 days, and III = 5/6 days). In each sampling time, three biological replicates were collected, containing 100 guts each, totaling 18 libraries depleted in ribosomal RNA. Reads were quality-filtered and mapped against the Chinese JXGC Las strain and the Floridian strain UF506 for the analysis of the activity of Las genome and SC1, SC2, and type 3 (P-JXGC-3) prophages of the studied Las strain. Gene activity was considered only if reads of at least two replicates for each acquisition access period mapped against the selected genomes, which resulted in coverages of 44.4, 79.9, and 94.5% of the JXGC predicted coding sequences in Las I, Las II, and Las III, respectively. These genes indicate an active metabolism and increased expression according to the feeding time in the following functional categories: energy production, amino acid metabolism, signal translation, cell wall, and replication and repair of genetic material. Pilins were among the most highly expressed genes regardless of the acquisition time, while only a few genes from cluster I of flagella were not expressed. Furthermore, the prophage region had a greater coverage of reads for SC1 and P-JXGC-3 prophages and low coverage in SC2 and no indication of activity for the lysis cycle. This research presents the first descriptive analysis of Las transcriptome in the initial steps of the *D. citri* gut colonization, where 95% of Las genes were active.

## Introduction

“*Candidatus* Liberibacter asiaticus” (Las), a Gram-negative bacterium belonging to the α-proteobacteria group (Garnier et al., 1984; Jagoueix et al., 1994), is the main agent associated with huanglongbing (HLB) (Bassanezi et al., 2020). The Las vector insect is the Asian citrus psyllid (ACP) *Diaphorina citri* Kuwayama (Hemiptera: Psyllidae) (Capoor et al., 1967; Canale et al., 2017) that frequently acquires Las in areas surrounding healthy commercial groves, with the consequent psyllid dispersion causing primary infections (Bergamin Filho et al., 2016) resulting in economic loss due to impacted production (Bassanezi et al., 2020). Owing to the importance of the disease and because there are no curative methods to control HLB, the bacterium and the psyllid are considered to be the main pests in citriculture worldwide (Miranda and Ayres, 2020).

The psyllid may acquire Las from symptomatic plants (Hung et al., 2004; Pelz-Stelinski et al., 2010; Canale et al., 2017; Lopes and Cifuentes-Arenas, 2021) as well as from asymptomatic citrus plants (Lee et al., 2015). Las transmission by psyllids requires a latent period of 16 to 18 days (Canale et al., 2017) and its relationship with the psyllid is persistent and propagative (Hung et al., 2004; Inoue et al., 2009; Ammar et al., 2011a; Canale et al., 2017). The acquisition of bacteria by the psyllid has direct implication on its transmission efficiency and consequently on its dissemination (Inoue et al., 2009; Pelz-Stelinski et al., 2010; Canale et al., 2017). The gut represents the first cell barrier that the Liberibacter needs to cross inside the psyllid before accessing the hemolymph to migrate and infect salivary glands, from where it will be inoculated into plants. Gene expression studies showed a pattern of response in the gut and salivary glands of *D. citri* to Las infection (Kruse et al., 2017; Liu et al., 2020). There are exploratory studies that report transcriptional modifications in plants due to the presence of Las in *Catharanthus roseus* (Liu et al., 2019) and in leaves (Fu et al., 2016; Hu et al., 2017) and fruits of *Citrus* spp. (Martinelli et al., 2012).

The transcriptional analysis of Las is more commonly assessed *via* RT-qPCR in psyllids and in citrus, with modifications in the expression of genes related to the regulation of transcription, transport, secretion, flagellar assembly, metabolic pathways, and resistance to stress, by the assessment of a group of selected genes (Yan et al., 2013; Prasad et al., 2016; Thapa et al., 2020) or associated with functional studies (Fleites et al., 2014; Jain et al., 2015; Li et al., 2017). Sequencing of cDNA libraries *via* RNA-Seq has been an important tool in studies of transcriptional modification in bacteria (Croucher and Thomson, 2010; Haas et al., 2012; Creecy and Conway, 2015). Transcriptome analysis of Las genome in citrus and dodder has shown a high correlation in gene expression in both hosts, expected since dodder was parasitizing citrus, and in both hosts, Las encounters similar nutrients and micro-environmental conditions (Li et al., 2021). A predicted model of metabolite usage was created for *Liberibacter crescens* growing in culture media and for Las in plant and psyllid hosts, using RNA-seq data (Zuñiga et al., 2020). A transcriptome analysis of *Bactericera cockerelli* indicated a high percentage of “*Ca.* L. solanacearum” (Lso) expressed genes, particularly of putative genes involved in translation and in post-translation modification, protein turnover, and chaperone functions (Ibanez et al., 2014). Here, “*Ca.* L. asiaticus” had 95% of genes mapped with reads in the *D. citri* gut, with pilins, flagella, translation, and cell wall biogenesis genes with marked expression. The Las prophage genes, with their high diversity in Las populations, were mapped mostly to SC1 and type 3 prophages, in both late and early genes, without indication of an active lytic cycle. The transcriptional mapping analysis of Las genes in the gut of *D. citri* that fed in Las-infected citrus was performed in this study, evidencing the expression of almost all genes of Las as a manner to generate knowledge in the initial phase of the Las infection in the psyllid.

## Materials and Methods

### Obtention of Samples

The stock colony of Las-free *D. citri* was maintained in cages in *Murraya paniculata* L. (Jack) plants, as described by Carmo-Souza et al. (2020). Adults from 7 to 10 days were selected for the Las acquisition step.

Nursery plants of sweet orange [*Citrus* × *sinensis* (L.) Osbeck grafted on “Rangpur” lime (*C*. × *limonia* Osbeck)] were maintained in 4-L bags in a greenhouse and grown in decomposed pine bark substrate (Plantmax citrus, Eucatex, Paulínia, SP, Brazil). Las infection was confirmed by qPCR (Li et al., 2006), and plants presented symptoms of blotchy mottle. Healthy plants of the same age and under similar growing conditions were used as negative control. The Las isolate employed in this study had a profile of SSR markers and prophages identical to Las 9PA isolate (Silva et al., 2019, 2021).

Plants underwent drastic pruning and were maintained under controlled conditions of temperature of 26°C ± 2°C, relative humidity of 70%, and photoperiod of 14/10 h of light/dark for shoot development and experimentation. Adult psyllids were confined and kept on citrus shoots at the vegetative stage V2/V3 of both healthy and Las-infected sweet orange plants (Cifuentes-Arenas et al., 2018; Lopes and Cifuentes-Arenas, 2021). Prior to the confinement of the insects, shoots were sampled to confirm the presence of Las (Li et al., 2006).

Dissection of psyllids for gut sampling was carried out after different acquisition access periods of 24, 48, 72, 96, 120, and 144 h, equivalent to 1- to 6-day intervals of acquisition. Psyllids from each period were removed from plants and dissected in 70% ethanol with 1% diethyl pyrocarbonate (DEPC) with the aid of a needle under stereoscopic microscope. Three repetitions were performed for each acquisition period in healthy or infected plants, each comprising 100 guts. Guts were immediately transferred to 100 μl of RNA Later (Invitrogen/ThermoFisher Scientific, Waltham, MA, United States, EUA) in ice and then immediately stored at −80°C.

### Extraction of Total RNA From Psyllid Gut Samples, Detection of the Presence of “*Candidatus* Liberibacter asiaticus,” and Treatment With DNase

Extraction of total RNA from dissected psyllid gut samples was performed using the SV RNA Isolation System (Promega, Madison, WI, United States) kit. Samples were resuspended in 30 μl of nuclease-free water and were assessed by spectrophotometry in NanoDrop V 3.8.1 (ThermoFisher Scientific) in relation to total RNA concentration.

The detection of the presence of Las was carried out prior to treating RNA samples with DNase by qPCR (Li et al., 2006), monitoring the presence of Las 16S rDNA (Table 1) and of the *wingless* gene (Table 1) for *D. citri* DNA (Manjunath et al., 2008) using hydrolysis probes (PathID qPCR Master Mix, Ambion/ThermoFisher Scientific; Primers and probes by Macrogen, Seoul, South Korea). The target sequence threshold was manually adjusted in the StepOnePlus software version 2.3 (ThermoFisher Scientific). The qPCR was carried out in duplicate and samples were considered positive for the presence of Las when Ct values were equal to or lower than 35.0 and negative when greater than that value. The presence of *D. citri* DNA produces Ct values equal to or lower than 36.0. Psyllid DNA samples with and without Las were used as positive and negative controls, respectively, besides water control (non-template control).

**TABLE 1:** Primers and probes to detect 16SrDNA from “*Candidatus* Liberibacter asiaticus” and *wingless* gene from *Diaphorina citri* using qPCR.

Primer/Probe name	Nucleotide sequences (5′ to 3′) and modifications (probes)	References
HLBas	TCGAGCGCGTATGCAATACG	Li et al., 2006
HLBr	GCGTTATCCCGTAGAAAAAGGTAG	
HLBp	FAM-AGACGGGTGAGTAACGCG-BHQ1	
DCF	TGGTGTAGATGGTTGTGATCTGATGTG	Manjunath et al., 2008
DCR	ACCGTTCCACGACGGTGA	
DCP	HEX-TGTGGGCGAGGCTACAGAAC-BHQ1	

Selected total RNA samples were treated with Turbo^TM^ DNase (2 U/μl) (Ambion/ThermoFisher Scientific). The confirmation of the DNase action to cleave residual DNA was validated by qPCR with the *wingless* gene for *D. citri* (Manjunath et al., 2008).

### Enrichment of mRNA in Samples and Quality Assessment for Sequencing

At this step, samples were grouped into pools: H = Healthy citrus or Las = Las-infected citrus sources. H I and Las I = 1 and 2 days of feeding on citrus; H II and Las II = 3 and 4 days; H III and Las III = 5 and 6 days. Each period and source had three biological repetitions. Next, samples were subjected to mRNA enrichment with the Ribo-Zero Gold rRNA Removal Kit (Illumina, San Diego, CA, United States). Two microliters of each sample was used to determine mRNA concentration and quality in the Synergy Multi-Detection reader (Biotec Synergy Winooski, EUA; software Gen5). Samples with total mRNA concentrations between 80 and 200 ng were used for metatranscriptome sequencing.

### cDNA Sequencing *via* Illumina Platform

Samples enriched in mRNA were subjected to the cDNA library preparation protocol TruSeq RNA Library Prep Kit (Illumina), according to the paired-end (2 × 100 bp) strategy that consists in fragmenting the mRNA-enriched RNA into sequences of 200 nucleotides, followed by binding with random primers for reverse transcription and building of cDNA libraries. The cDNA was synthesized and purified through magnetic beads and washings with ethanol. Next, ends were repaired and adenosines were added to the 3′ ends of each fragment. Then, adaptors were connected and samples were subjected to PCR amplification. Sequencing of cDNA libraries was carried out on the Illumina HiScanSQ platform (Illumina), at the Centro Multiusuário de Biotecnologia Agrícola of the Departamento de Zootecnia at ESALQ/USP (Piracicaba, SP, Brazil). Readings were obtained from triplicates of the six treatments, for periods I, II, and III for healthy and Las-infected adults, totaling 18 libraries.

### Sequencing Analysis and Transcriptome Assembly

Readings obtained from sequencing cDNA libraries were analyzed with the FastQC software (Andrews, 2010) for quality assurance. Remaining adaptors were removed and sequences were trimmed according to the SLIDINGWINDOW 4:22 parameter for the start (LEADING:3) and end (LEADING:3) of readings, which determined the minimum quality score (22) for each group of four nucleotides analyzed in the Trimmomatic 0.36 software (Bolger et al., 2014). Sequences below 25 nucleotides were excluded.

The analysis of Las transcripts abundance in the ACP gut was carried out by counting the number of fragments per kilobase million (FPKM) through the calculation of log2(FPKM) of the reads present in at least two out of the three sequenced libraries for each of the acquisition access periods (Las I, Las II, and Las III). Las strain JXGC (NZ_CP019958; Zheng et al., 2018) was the reference for mapping and counting of reads in Las genome and consequently for determining the transcribed genes. The draft genome of Las strain 9PA from Brazil was not used due to the presence of several gaps in the genome assembly (Silva et al., 2021). Reads mapped to ribosomal RNA, pseudogenes, and tRNAs were disregarded, the latter due to their size. Additionally, in the analysis of SC1 and SC2 prophages, the sequence of the strain Las UF506 (HQ377374.1; Zhang et al., 2011) and that of prophage type 3 (P-JXGC-3) of Las JXGC (KY661963) were used. Those analyses employed the following parameters: mismatches cost: 2; insertion cost: 3; deletion cost: 3; length fraction: 0.8; similarity fraction: 0.8; strand specific: both; maximum number of hits for a read: 2, and were carried out in the CLC Genomics v. 21.0 software (Qiagen, Hilden, Germany).

The functional annotation of transcripts (Cluster of Orthologous Groups of proteins—COGs), generated through mapping genes of Las, was carried out (Tatusov et al., 2000). Three reference genomes were used, of which two were α-proteobacteria: “*Ca.* L. solanacearum” (strain CLso-ZC1), *Rhizobium leguminosarum* bv. viciae (strain 3841), and *Escherichia coli* (O157:H7 strain Sakai). Graphs were plotted with GraphPad Prism version 8.0.0 (Windows GraphPad Software, San Diego, CA, United States) and the Venn diagram was plotted using “*Draw Venn Diagram*”^1^.

## Results

### Presence of “*Candidatus* Liberibacter asiaticus” in Gut Samples of Psyllid *D. citri*

The qPCR analysis of citrus shoots used as food by psyllids confirmed the presence of Las exclusively in infected plants, with an average cycle threshold (Ct) of 20.7 (standard error ± 3.4). The presence of the psyllid DNA was detected in all gut RNA samples, with an average Ct for the *D. citri wingless* gene of 30.7 (Table 2), corroborating the need for treatment with DNase before cDNA synthesis. The DNA presence, however, enabled the monitoring of the Las DNA presence by qPCR as a way to validate the presence of the bacterium in the dissected guts of *D. citri* before transcriptome analysis. The presence of Las was detected only in RNA samples from guts of insects that fed on shoots of Las-infected plants (Table 2). In the Las I and Las II libraries, the mean Ct-value for the Las gene 16S rRNA was 30.6 and 30.8, respectively, whereas in Las III libraries, the Ct-value was 27.3. For gut samples of psyllids confined in healthy plants (libraries H I, II, and III), Las DNA was not detected.

**TABLE 2:** Spectrophotometrically measured RNA concentration (ng/μl) and quality (260/280) and detection of *Diaphorina citri* (DC) and “*Candidatus* Liberibacter asiaticus” (Las) (HLBaspr) DNA by qPCR in RNA samples prepared from *D. citri* gut, prior to DNase treatment and library setup.

AAP	Sample	RNA		qPCR Ct ^a^	
			
		ng/μl	260/280	DC ^b^	HLBaspr ^c^
I = 1/2 days	H I_1	59.06	2.06	31.11 ± 0.77	nd
	H I_2	68.86	2.16	29.91 ± 0.20	nd
	H I_3	46.59	2.07	30.44 ± 0.79	nd
	Las I_1	56.20	2.03	30.25 ± 0.15	32.18 ± 2.72
	Las I_2	56.85	2.10	30.77 ± 0.43	30.68 ± 0.54
	Las I_3	65.10	2.05	29.20 ± 0.12	28.99 ± 0.87
II = 3/4 days	H II_1	69.69	2.12	31.91 ± 1.27	nd
	H II_2	58.69	2.12	29.39 ± 1.87	nd
	H II_3	57.89	2.13	29.46 ± 1.20	nd
	Las II_1	74.56	2.04	30.52 ± 1.49	29.69 ± 1.53
	Las II_2	48.18	2.08	31.17 ± 0.21	32.24 ± 2.08
	Las II_3	65.88	2.13	33.71 ± 0.04	30.45 ± 0.68
III = 5/6 days	H III_1	68.21	2.01	30.09 ± 0.16	nd
	H III_2	58.44	2.07	30.90 ± 0.11	nd
	H III_3	46.42	2.07	32.50 ± 1.71	nd
	Las III_1	51.26	2.14	28.96 ± 0.85	26.18 ± 2.10
	Las III_2	45.92	2.02	30.10 ± 0.80	26.48 ± 0.02
	Las III_3	51.08	2.04	31.86 ± 0.77	29.40 ± 0.69

**Diaphorina citri* gut samples dissected from psyllids reared in either healthy (H) or Las-infected citrus plants (Las) for increasing (I, II, and III) acquisition access periods (AAP) of time. ^*a*^Ct, Cycle threshold, mean from duplicate value ± standard error; nd, not detected. ^*b*^DC = qPCR for *D. citri* wingless gene detection (Manjunath et al., 2008). ^*c*^HLBaspr = qPCR for “Ca. L. asiaticus” 16S rDNA detection (Li et al., 2006).*

### Mapping of Reads of “*Candidatus* Liberibacter asiaticus”

The average percentage of mapped reads in the “*Ca.* L. asiaticus” genome in healthy samples (H I, II, and III) was 0.01, 0.00, and 0.02, respectively, whereas that percentage in libraries set up from guts of psyllids that fed on Las, Las I, II, and III-infected plants was 0.07, 0.16, and 0.53, respectively (Table 3).

**TABLE 3:** Transcript read mapping against “*Candidatus* Liberibacter asiaticus” (Las) JXGC strain (NZ_CP019958.1) from sequenced libraries prepared with enriched mRNA from *Diaphorina citri* gut samples, reared either in healthy (H) or in Las infected-citrus shoots (Las), for increasing (I, II, and III) acquisition access periods (AAP).

AAP	Library	Input reads	Mapped paired pairs (%)
I = 1/2 days	H I_1	14,662,250	0.00
	H I_2	17,002,637	0.01
	H I_3	16,119,271	0.01
	Mean	15,928,053	0.01
	Las I_1	16,809,192	0.06
	Las I_2	16,066,925	0.19
	Las I_3	14,342,296	0.09
	Mean	15,739,471	0.07
II = 3/4 days	H II_1	15,288,196	0.00
	H II_2	17,110,015	0.01
	H II_3	17,831,678	0.00
	Mean	16,743,296	0.00
	Las II_1	15,083,210	0.09
	Las II_2	16,125,467	0.03
	Las II_3	14,389,488	0.36
	Mean	15,199,388	0.16
III = 5/6 days	HIII_1	16,064,435	0.01
	HIII_2	15,614,261	0.04
	HIII_3	15,142,440	0.01
	Mean	15,607,045	0.02
	Las III_1	17,908,705	0.40
	Las III_2	16,163,569	0.86
	Las III_3	16,311,141	0.33
	Mean	16,794,472	0.53

**FIGURE 1:**
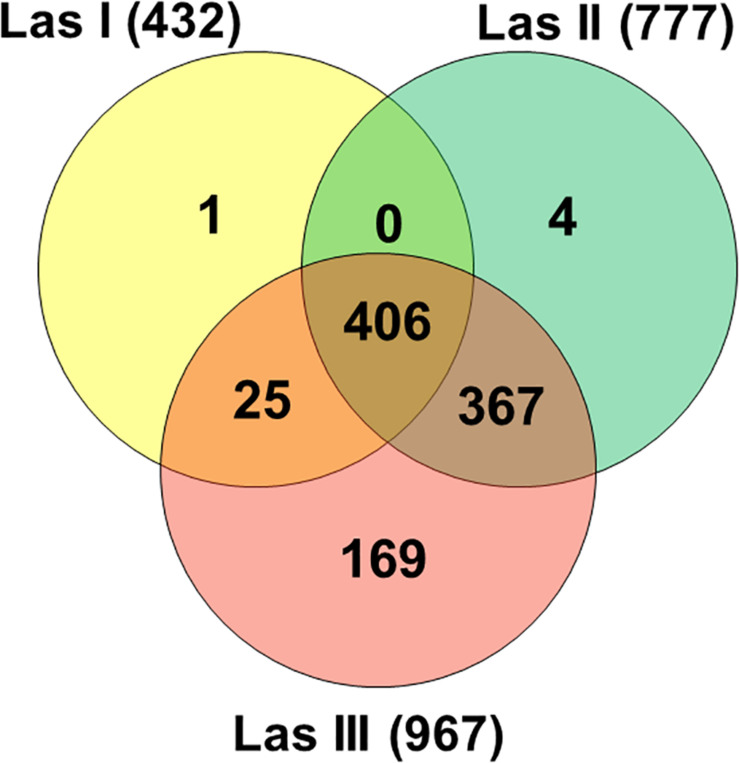
Venn diagram of “*Candidatus* Liberibacter asiaticus” genes with mapped reads (strain JXGC, NZ_CP019958.1) in *Diaphorina citri* gut samples after acquisition access periods of 1/2 days (Las I), 3/4 days (Las II), or 5/6 days (Las III).

Reads in Las I libraries mapped against 432 genes of the Las JXGC reference genome, whereas reads in Las II and Las III libraries mapped against 777 and 967 genes, respectively (Figure 1). Out of the genes predicted in the Las JXGC isolate (Zheng et al., 2018), 972 had reads mapped in at least one of the three acquisition access periods (Las I, II, or III) in the gut of psyllids that fed on shoots of Las-infected plants. The transcriptome of all libraries in the three periods assessed corresponds to mapping of 95% of the genes predicted in Las. Out of that total, 406 or 41.8% of mapped genes were shared among libraries from all acquisition periods, whereas 367 genes were mapped only to reads in the Las II and Las III libraries (Figure 1). Reads in the Las I library were the only ones to map against the gene B2I23_RS05235 that codes a YdaU family protein (unknown function with domain DUF1376), showing the specific expression of this gene at the initial stage of the acquisition process. Four other genes (B2I23_RS00005, a prophage helicase; B2I23_RS00270; B2I23_RS02880; and B2I23_RS05570, coding three hypothetical proteins) were mapped only to reads in the Las II library. In the libraries Las III, 169 genes were mapped exclusively in this stage, with COG distribution equivalent to the whole library set. Into that group were 57 hypothetical protein coding genes, two of the three full-length β-subunits of ribonucleotide reductase (RNR) (B2I23_RS03685 and B2I23_RS04305), and 12 genes from the flagellar clusters (Supplementary Table 1). Only 51 of the genes predicted in the Las strain JXGC had no mapped reads in the transcriptome of the gut of infected adult psyllids, showing that these genes are not expressed in the vector gut during the infection process. These comprise two genes coding for the short forms of the β-subunit of RNR B2I23_RS00035 and B2I23_RS04515, one containing the VRR-NUC (B2I23_RS05600) domain, two containing the DUF59 domain (B2I23_RS01860 and B2I23_RS03885), one containing the DUF2815 domain (B2I23_RS05410), one ATPase (B2I23_RS02840), one nuclease (B2I23_RS05420), one primase (B2I23_RS05400), and 35 genes that code hypothetical proteins (Supplementary Table 2).

**FIGURE 2:**
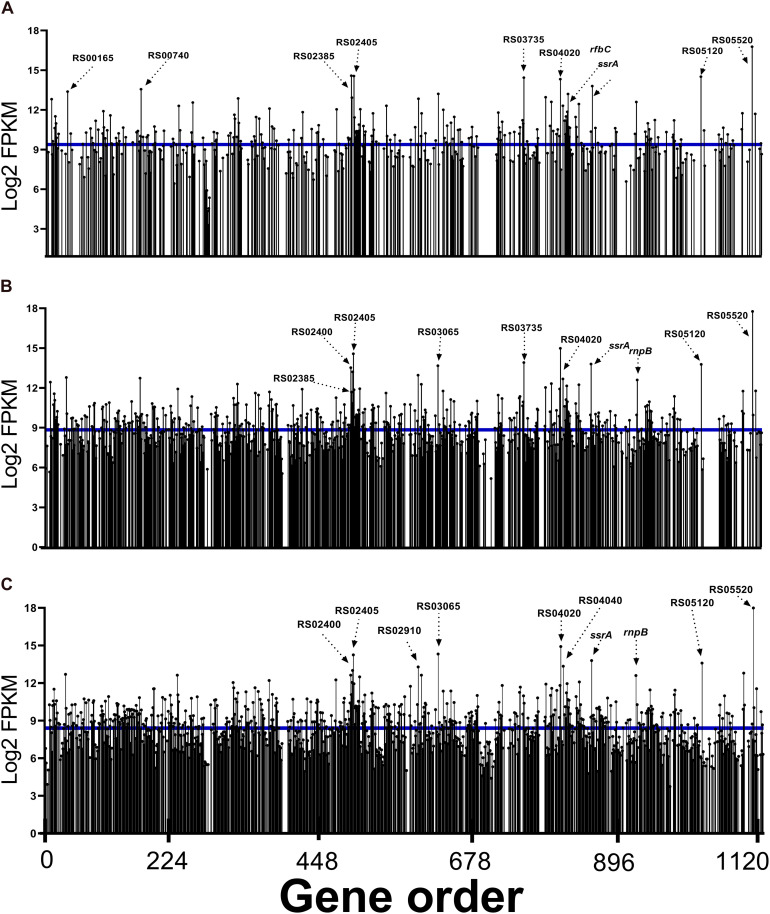
Read abundance mapped to “*Candidatus* Liberibacter asiaticus” genes (reference strain JXGC, NZ_CP019958.1) expressed in gut samples from *Diaphorina citri* after acquisition access periods of 1/2 days (Las I) **(A)**, 3/4 days (Las II) **(B)**, or 5/6 days (Las III) **(C)**, mean log2FPKM.

In the analysis of read abundance for each library and its distribution along the JXGC reference genome, an average of 42.2% of the genes predicted in the genome was obtained for Las I (Figure 2), and in the Las II and Las III libraries, coverage was 76 and 94.5%, respectively (Figure 2). Although the number of genes with mapped reads increased considerably according to the period of time the psyllid remained feeding on the Las-infected plant (Las III > Las II > Las I), the average log2(FPKM) value was slightly reduced from 9.38 in Las I to 8.84 and 8.41 in Las II and Las III, respectively (Figure 2). Only in the Las III library is there a greater number of mapped genes with values higher than the average.

When the top 10 genes with a greater number of reads in each period are analyzed, three are annotated as coding hypothetical proteins: B2I23_RS05520, B2I23_RS05120, and B2I23_RS04020 mapped in all periods (Figure 2).

The transcription of genes *flp1* (B2I23_RS02385), *flp4* (B2I23_RS02400), and *flp5* (B2I23_RS02405) annotated as “*Flp family type IVb pilin*” was significant in the Las I, II, and III libraries. Besides these three genes, *flp2*, *flp3*, and *flp7* were also mapped (B2I23_RS02390; RS02395 and RS02410). Reads for B2I23_RS03065, whose annotation is for a membrane protein (“*porin family protein*”), were also expressive.

Reads for the gene B2I23_RS03735 (“*NADH-quinone oxidoreductase subunit C*”) were present among the 10 genes with greater representativeness in the Las I and Las II libraries. B2I23_RS04070 (*rfb*C) that codes the dTDP-4-dehydrorhamnose 3,5-epimerase involved in the synthesis of rhamnose-containing polysaccharides, such as lipopolysaccharides, is highly expressed in Las I. An ATP-dependent Clp protease ATP-binding subunit, *clpX* (B2I23_RS00740), was mapped in Las I as one of the most expressed genes (Figure 2). Additionally, all genes coding for components of the protease Clp family were expressed in Las III: *clpA* (B2I23_RS00185), *clpS* (B2I23_RS00190), endopeptidase La (B2I23_RS00735), *clpP* (B2I23_RS00745), *hslU* (B2I23_RS01895), *hslV* (B2I23_RS01900), lon peptidase (B2I23_RS02370), and *clpB* (B2I23_RS03845) (Supplementary Table 3).

The other genes mapped with a representative number of reads are involved in ribosome metabolism, such as the transfer-messenger RNA–*ssrA* (B2I23_RS04260), *rnpB*–RNAse P (B2I23_RS04605), and ribosomal proteins *rpmJ* (B2I23_RS00165) and *rpsT* (B2I23_RS02910) genes. The chaperone gene for the “*cold shock domain-containing protein*” (B2I23_RS04040) is among the most expressed in Las III.

**FIGURE 3:**
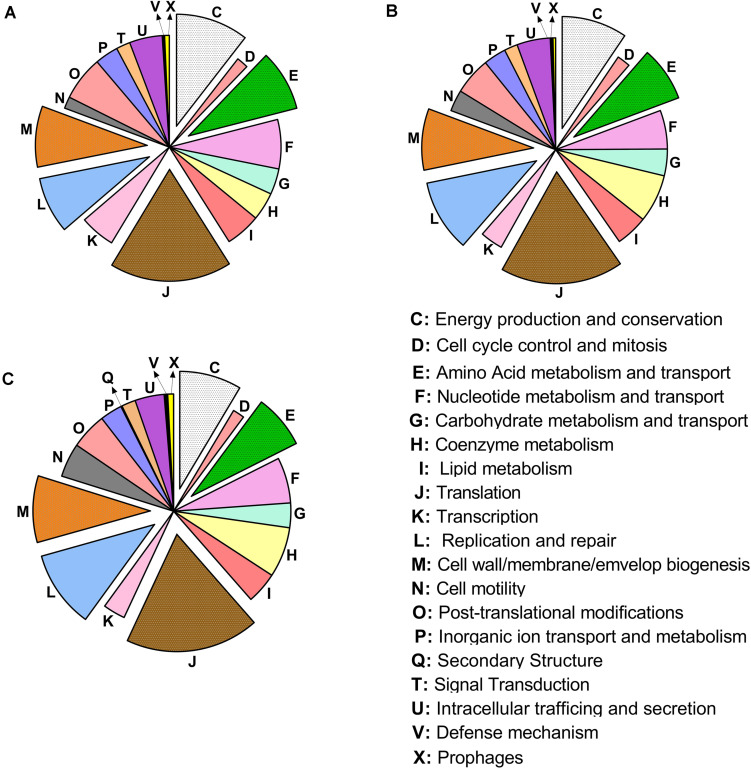
Distribution of the cluster of ortholog groups in “*Candidatus* Liberibacter asiaticus” (reference strain JXGC, NZ_CP019958.1), measured as mapped reads in *Diaphorina citri* gut after acquisition access periods of 1/2 days (Las I) **(A)**, 3/4 days (Las II) **(B)** or 5/6 days (Las III) **(C)**.

The classification of genes mapped in the COGs (Tatusov et al., 2000) identified a greater number of genes mapped in the COGs translation (J), replication and repair (L), cell wall/membrane biogenesis (M), energy production (C), and amino acid transport and metabolism (E) (Figure 3).

**FIGURE 4:**
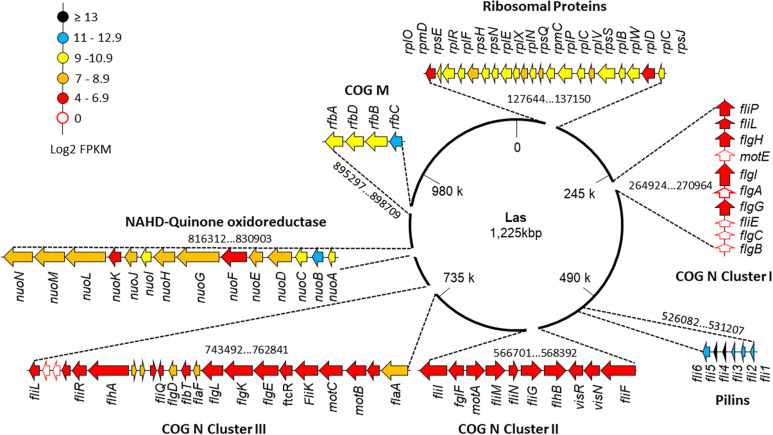
Gene expression level along “*Candidatus* Liberibacter asiaticus” chromosome of selected clusters of genes. Flagellar clusters (COG N: Clusters I, II, and III), “Flp family type IVb pilins” (COG U), transferases (COG M), ribosomal proteins, and NADH-quinones genes are individually shown and their position in the chromosome is indicated. Read abundance (log2FPKM) taken from *Diaphorina citri* gut library from the acquisition time 5/6 days (Las III) mapped in strain JXGC (NZ_CP019958.1).

When only Las III libraries were analyzed, Clusters I, II, and III that code flagellar proteins (COG N) with average values of log2(FPKM) of 5.53, 6.27, and 6.11, respectively (Figure 4), stand out. The genes *motE*, *flgA*, *fliE*, *flgB*, and *flgC* were not mapped (Supplementary Table 1). The sequences predicted for *pilins* (COG: U) present average values of log2(FPKM) of 12.49, above the average value of Las III. A region with genes for proteins involved in the lipopolysaccharide synthesis (COG: M) presented values of log2(FPKM) of 10.8, also above the average in Las III. The region that includes 21 out of the 54 ribosomal proteins annotated in the Las genome, located in the region 128 kbp to 137 kbp, and others where 14 subunits of NADH-quinone oxidoreductases are annotated in the region 816 kbp to 829 kbp (Figure 4), presented average log2(FPKM) values of 9.0 and 8.4, respectively. The list with FPKM and log2(FPKM) values for all mapped genes from the Las JXGC strain in each library is available as Supplementary Table 3.

### Mapping of Las Prophages SC1, SC2, and P-JXGC-3 in the Gut of *D. citri*

**FIGURE 5:**
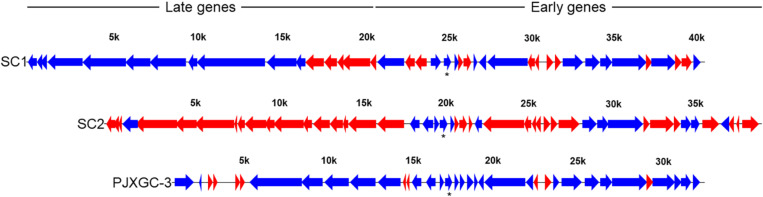
Read mapping against “*Candidatus* Liberibacter asiaticus” (Las) SC1 (type 1) and SC2 (type 2) prophages from Las UF506 (HQ377374) and P-JXGC-3 (type 3) prophage from Las JXGC (KY661963). Blue arrows indicate genes mapped in at least two libraries while red arrows had no reads mapped. Asterisk is the Las genes with the highest number of mapped reads. Expression level taken from *Diaphorina citri* gut library from the acquisition time 5/6 days of rearing in Las-infected citrus (Las III).

Reads from Las-infected libraries mapped against the genomes of prophages SC1 (UF506) and type 3 (P-JXGC-3) (Figure 5). In the widely conserved region shared between both prophages, known as early genes and also shared with SC2, the high similarity of gene sequences does not allow for differentiation as to the presence of reads between prophages, except in divergent regions. There were no reads mapped in the SC1/type 3 genes: SC1_gp175/PJXGC_gp25 and SC1_gp185/PJXGC_gp26, both coding hypothetical proteins, and in SC1_gp215/PJXGC_gp32, an endonuclease (VVR-NUC). The other shared genes were mapped, while a few divergent genes were not mapped (Figure 5). The highest number of mapped reads among all libraries was for the hypothetical protein PJXGC_gp17 gene (B2I23_RS05520)/SC1_gp135 (Figure 2).

In prophage late region, read coverage was 68.75% in the SC1 genes and 60% in P-JXGC-3 (Figure 5). In the prophage SC1, the following genes had reads mapped (genes without indication are hypothetical proteins): SC1_gp005, SC1_gp010, SC1_gp015, SC1_gp025 (tail fiber protein), SC1_gp030, SC1_gp035 (endolysin), SC1_gp045, SC1_gp050, SC1_gp060 (colicin IA), SC1_gp080, and SC1_gp085 (major tail subunit). The following genes were not mapped: SC1_gp090 (major capsid protein), SC1_gp095, SC1_gp100, SC1_gp105 (head-to-tail joining protein), and SC1_gp110 (holin). In P-JXGC-3, only the genes PJXGC_gp01, PJXGC_gp02, and PJXGC_gp07 to PJXGC_gp10 that code subunits R and S of the deoxyribonuclease type I site-specific (HsdR) and two subunits of the SAM-dependent DNA methyltransferase, respectively, were mapped. From the SC2 prophage, only SC2_gp020 was exclusively mapped.

## Discussion

*Diaphorina citri* acquires Las during feeding on infected plants (Hung et al., 2004; Inoue et al., 2009; Pelz-Stelinski et al., 2010), while transovarial transmission of Las to vector offspring is reported as low or absent (Hung et al., 2004; Pelz-Stelinski et al., 2010). The obtention of *D. citri* gut samples with Las requires manipulation of individuals to dissect them while keeping not only the integrity of the organ but also the quality and amount of mRNA necessary, in particular from the bacterium, to reveal genes whose transcription does occur in the psyllid gut. *D. citri* feeding on young citrus flushes infected by Las, under proper environmental conditions as those used in this study, generates high rates of infective psyllids, also able to transmit Las at high rates as a consequence of the high bacterial titer in the psyllid (Lopes and Cifuentes-Arenas, 2021). Psyllids that fed on Las-infected citrus shoots for a period of 5 to 6 days had 10 times more Las in their guts than those that fed for shorter periods of up to 4 days. The employed methodology enabled us to assess the Las transcriptome in the gut lumen and in association with gut epithelium from *D. citri*. Meanwhile, lower Ct-values for Las 16SrDNA were observed for the longer feeding time and higher Las read mapping was found in those libraries (Las III > Las II > Las I). For the longer feeding time (Las III, of 5 and 6 days) assessed in Las-infected plants, 0.53% of total reads mapped against Las reference genome JXGC. This value falls in the range observed by other authors, such as the mapping of 0.50% of transcriptome reads of the whole body of *B. cockerelli* against the Lso genome (Ibanez et al., 2014). Transcriptome analysis of Las was comparatively carried out in citrus and in dodder, with read mapping ranging from 0.11% to 1.44%, respectively (Li et al., 2021), while in adventitious citrus roots in culture media and in alimentary canals of *D. citri*, 0.28% to 2.51% of reads mapped to Las genome (Zuñiga et al., 2020). In contrast, only 0.01% of reads obtained from infected grapevine mapped against the flavescence dorée phytoplasma (Abbà et al., 2014). Las DNA detection in relation to host DNA is higher in psyllids than in citrus (Selvaraj et al., 2018), and this favors the obtention of Las nucleic acid from psyllids for metagenomic (Duan et al., 2009; Lin et al., 2013) and metatranscriptomic (Zuñiga et al., 2020) analysis.

The gut represents the first cell barrier that Las has to cross to systemically infect the psyllid body (Ammar et al., 2011a) and Las achieves high titer in this organ (Ammar et al.,2011a,b; Ghanim et al., 2017), though most of psyllid organs become infected (Ammar et al.,2011a,b). The transmission efficiency of Las by psyllids is related to the ability of the bacteria to multiply in the psyllid body (Ukuda-Hosokawa et al., 2015), as well as their ability to cross internal barriers in the ACP body, particularly the psyllid gut, hemolymph, and salivary glands (Ammar et al.,2011a,b; Ammar et al., 2016; Cicero et al., 2017; Ghanim et al., 2017). In this regard, the transcriptional status of Las in the gut is critical for psyllid infection and colonization. Reads from psyllids fed on healthy citrus plants (H I, II, and III) have, in addition to hits of psyllids sequences, reads with sequences similar to Las from endosymbiont bacteria, such as in the conserved regions from bacterial ribosomal genes. Depletion of ribosomal RNA prior to library construction allowed mRNA enrichment with the concomitant obtention of a representative number of reads (0.53%) with a Las gene expression coverage of 95%. While part of the reads still mapped to ribosomal genes, as observed in the RNA-Seq of Lso (Ibanez et al., 2014), depletion of such abundant sequences is essential to transcriptomic studies of eukaryote-associated bacteria (Kumar et al., 2015). *D. citri* has a rich microbial flora, composed of primary syncytium endosymbionts, “*Ca*. Carsonella ruddi” and “*Ca*. Profftella armature” (Nakabachi et al., 2013), secondary intracellular symbionts (Subandiyah et al., 2000; Guidolin and Cônsoli, 2013), and a range of extracellular bacteria (Kolora et al., 2015). The gene *lys*E from Las (B2I23_RS04390) codes for a protein whose function is predicted in amino acid transport and metabolism. That gene was acquired from “*Ca*. P. armatura” (Nakabachi et al.,2013a,b), indicating not only horizontal gene transference but also similarity between gene sequences from endosymbiotic bacteria with Las in the psyllids. Besides primary endosymbionts, *D. citri* populations in particular from Brazil have fixed infections by *Wolbachia* (Guidolin and Cônsoli, 2013; Dossi et al., 2014). Average incidence of Las in *D. citri* was 65% in São Paulo State/West Minas Gerais State in Brazil (Wulff et al., 2020).

Plant pathogenic Liberibacters infect a range of hosts of economic importance (Garnier et al., 1984; Teixeira et al., 2008; Bové, 2006; Liefting et al., 2008; Munyaneza et al., 2009; Thapa et al., 2020), share features that render their study cumbersome, such as growth restricted to the phloem of infected plants, and are well-adapted to psyllid vectors. The majority of bacteria with reduced genome sizes, as is the case of Liberibacters (Duan et al., 2009; Lin et al., 2011; Wulff et al., 2014; Lin et al., 2015), have lost genes underlying the biosynthesis of compounds readily available from the host, as well as regulatory elements such as sigma factors (Konstantinidis and Tiedje, 2004). *Ca*. Liberibacter americanus (Lam) is also associated with HLB symptoms and have *D. citri* as vector, although currently is rare to find in the field (Teixeira et al., 2008; Bassanezi et al., 2020). Genes absent in Lam (Wulff et al., 2014) but found in Las were expressed in the psyllid gut. A relation that can be seen in reduced genomes with adaptive pressure and evolutive process is the loss of redundant and non-functional gene sequences (Mira et al., 2001; Sällsträm and Andersson, 2005), a factor that certainly renders these Liberibacters highly host-dependent for growth. *L. crescens* is the only Liberibacter available in axenic culture, which makes it suitable to functional studies and growth assays (Leonard et al., 2012; Jain et al., 2015; Jain et al., 2017b; Cruz-Munoz et al., 2019; Zuñiga et al., 2020). In Las strains and other Liberibacters that have their genomes sequenced, type II secretion system, avr and hrp genes, plant cell wall degrading enzymes, and several known virulence determinants also found in other plant pathogenic bacteria are absent (Duan et al., 2009; Lin et al., 2011; Wulff et al., 2014; Lin et al., 2015) but potential Las effectors (Thapa et al., 2020) were expressed in the psyllid gut in our analysis, except for CLIBASIA_04530.

The greater resolution power provided by the transcriptome analysis as compared to RT-qPCR might be used to link diverse cellular processes to gain understanding in active metabolic pathways for Las (Zuñiga et al., 2020). The process of host invasion and colonization by pathogens, leading to multiplication and increased titer, requires gene expression for the synthesis of proteins from housekeeping functions and energy production. A high number of reads mapped to genes coding for ribosomal proteins, NADH-quinone oxidoreductases, and genes for lipopolysaccharides synthesis, collectively indicating metabolic activity of Las during gut infection in *D. citri*. B2I23_RS03735 codes for subunit C of NADH-quinone oxidoreductase (NuoC). Subunits NuoA to NuoN form the core enzyme of complex I for the bacterial respiratory chain (Spero et al., 2015). The *nuo*C gene was mapped as one of the most expressed in Las I and Las II, and the 14 genes of the complex I subunits were expressed in Las II and Las III. In most bacterial clades, *nuo*A to *nuo*N are expressed as a polycistronic operon (Spero et al., 2015). Yan et al. (2013) assessed the expression of *nuo*A from this operon and found higher expression in psyllids than in plants. The expression of genes from the complex I by Las during gut colonization indicates that NADH reoxidation maintains the redox state of the cell (Spero et al., 2015).

Overall, 35 genes coding for hypothetical proteins were not mapped in Las in the psyllid gut, whereas 44 genes were not expressed in Lso in the *B. cockerelli* body (Ibanez et al., 2014). A gene coding for a protein with a VRR-NUC domain was not expressed in either Liberibacter (CKC_RS01050 or B2I23_RS05600). Lack of expression of four out of the five genes preceding the insertion point of the type 3 prophage was observed in Las. Twenty-one of the genes not expressed in psyllid gut were also not expressed in citrus, while only three were not expressed in dodder (Li et al., 2021).

The Clp protease complex degrades unfolded proteins, transcription factors, and phage proteins, besides being involved in regulatory processes (Porankiewicz et al., 1999). The high read mapping to B2I23_RS00740 together with the expression of other genes from the protease complex indicates coordinated expression for proteolysis. B2I23_RS04070 codes for a dTDP-4-dehydrorhamnose 3,5-epimerase (RfbC) that together with RfbABD (Graninger et al., 1999) produces rhamnose containing-polysaccharide, such as those found in *Rhizobium* spp. (Ghosh and Maiti, 2016). All four genes (rfbABCD, B2I23_RS04070 to B2I23_RS04085) were highly expressed in all time periods, and additional genes for the lipopolysaccharide biosynthesis were gradually expressed from Las I to Las II, with all genes being expressed in Las III, indicating active synthesis of cell envelope components.

Chaperonins such as the cold shock domain-containing protein (B2I23_RS04040) and the hsp20 family protein (B2I23_RS02935) were highly mapped in Las genome in the *D. citri* gut. Lso in *B. cockerelli* also had high expression of the gene coding for a cold shock protein (CKC_03300), whose regulation is correlated with pathogenicity factors and adaptation to new environments (Ibanez et al., 2014; Mohamed et al., 2020). GroEL/GroES were expressed in all libraries. Not only the same cold shock domain gene but also chaperonins groEL/groES were among the 20 most expressed genes from Las in citrus (Li et al., 2021).

Six *flp* family type IVb pilin genes from Las were mapped in the gut libraries. Type IV pili is a virulence factor (Wairuri et al., 2012), and pilins exert a role during interaction with surfaces, notably during superficial adhesion, potentially being involved in biofilm formation. Las *flp3* is upregulated in whole psyllid bodies, while *visN* and *visR*, two LuxR-type transcriptional regulators, are downregulated (Andrade and Wang, 2019). In contrast to the expression profile observed in whole *D. citri* bodies, all six pilin genes were highly mapped in the gut, with *flp1*, *flp4*, and *flp5* among the most mapped genes. Las express four pilins in citrus (Li et al., 2021) and *flp1* has the highest number of mapped reads in contrast to that observed in psyllids (Andrade and Wang, 2019). Oppositely, *flp4* and *flp5* were not expressed in citrus (Li et al., 2021), but were in the psyllid gut. Both *visN* and *visR* were mapped in citrus, while in the psyllid gut, the expression of both regulators was only observed in the library Las III. Regulation of *flp3* by VisNR might allow Las to switch between forming biofilm and circulative status inside the psyllid hemocoel (Andrade and Wang, 2019). Lso establishes biofilm during gut infection of *B. cockerelli* (Cicero et al., 2016), and *L. crescens* forms biofilm *in vitro* (Naranjo et al., 2019). Whether Las also form biofilm during gut infection is expected but not shown yet.

Las genome codes for three flagellar clusters, even though flagella formation in Las is an open question (Andrade et al., 2019). While at clusters II and III all genes were expressed in Las III, cluster I had five genes not mapped, which might account for the absence of flagella in Las (Andrade et al., 2019), since *fliE*, *flgB*, and *flgC* are not expressed and form the proximal rod together with *flgF*, which, in turn, was expressed in Las III libraries. Flagella-like structures were found in *L. crescens* (Andrade et al., 2019) and superficial appendages were found in Lso (Cicero et al., 2016). Andrade et al. (2019) have shown a higher expression level of flagella genes in psyllids than in plants, which is in sharp contrast to what had been observed previously (Yan et al., 2013). The genes *motE* and *flgC* were not mapped in citrus also, in addition to *fliQ*, while *flg*A, *flg*B, and *fli*E had a low expression level (Li et al., 2021), also in agreement with Andrade et al. (2019) and the mapping analysis shown here.

Among the top 10 genes with higher reads mapped, three codes for hypothetical proteins (B2I23_RS04020, B2I23_RS05120, and B2I23_RS05520). In the first genome sequenced from Las, strain psy62, 26% of the coding sequences were annotated as hypothetical proteins (Duan et al., 2009). In the strain Las JXGC, the annotation of hypothetical proteins was reduced to 14% (Zheng et al., 2018), and from this, 72% were located in the genome from 687 kbp to 1219 kbp, a region where the above three hypothetical protein coding genes are located. Also, the P-JXGC-3 prophage is located in that region, whose 64% of genes code for hypothetical proteins, including the most mapped gene B2I23_RS05520. The presence and abundance of reads in genes from the prophage highlight the need to perform functional studies with such genes, as already carried out for some of the Las prophage genes (Fleites et al., 2014; Jain et al., 2015). Prophage presence stood as a hallmark of variation between Las genomes, and indeed the average nucleotide identity for Las isolates is between 99% to 100% (Thapa et al., 2020) except that larger differences among Las strains are located in prophage sequences (Duan et al., 2009; Zhang et al., 2011; Katoh et al., 2014; Zheng et al., 2018), mainly due to the presence or absence of either one of the prophages or parts of them. The lack or the presence of prophages can account for a large fraction of the variation among individuals within a bacterial species (Casjeans, 2003). Prophages in Las were first characterized as SC1 and SC2 (Zhang et al., 2011) and later a third prophage was described (Zheng et al., 2018). There are potential advantages to the prophage-harboring strains during host colonization. Prophages contribute to “*Ca*. Liberibacter spp.” diversity (Tomimura et al., 2009; Wulff et al., 2014; Jain et al., 2017a), while there is a small proportion of Las strains without prophages (Silva et al., 2019; Zheng et al., 2018, 2021). A higher read mapping was observed in genes from SC1 and P-JXGC-3 prophages and a few mapped to SC2. There are several genes potentially involved in lysogenic conversion in SC2 (Zhang et al., 2011), as well as in plant defense suppression, such as peroxidases coded by SC2_gp095 and SC2_gp100 (Jain et al., 2015). The absence of reads mapping to SC2, at least in the late region that is phage specific, in the current study is in accordance with the absence of a “standard” SC2 in Las strains from Brazil (Silva et al., 2019; Silva et al., 2021), as well as in the Las strain CoFLP1 from Colombia that is a strain from South America with a completely sequenced genome (Wang et al., 2021). Coding sequences for B2I23_RS05520 and B2I23_RS05445 are phage located and among the most mapped genes in Las in the gut of *D. citri*. While B2I23_RS05445 is exclusive from type 3 prophage, there might be a bias in read mapping for B2I23_RS05520, since this gene has identical copies in SC1 and type 3 prophages, both belonging to the early gene region of high similarity among Las prophages. Both genes were also highly mapped in Las transcriptome from citrus (Li et al., 2021). Genes whose products are involved in phage activity were expressed, such as SC1_gp115 (terminase), SC1_gp195 (exonuclease), SC1_gp210 DNA (polymerase A), and SC1_gp220 (helicase). The absence of reads mapped to SC1_gp090 (major capsid protein) and SC1_gp110 (holin) from Las while in the psyllid gut indicates no production of viral particles. Nevertheless, there is low similarity in the genomic sequence between SC1_gp090 (UF506) and Las strain 9PA (Silva et al., 2021) or the isolate CoFLP (Wang et al., 2021), and even though the Las strain from Brazil is not completely sequenced, the absence of the major capsid protein gene in the strain used is more likely, since neighboring genes are present as is the case of SC1_gp110 (holin) in that strain. The orthologous gene CD16_RS05495 that codes for a major capsid protein from Las strain A4 was expressed in citrus and dodder (Li et al., 2021), in agreement with phage particles being found in periwinkle (Zhang et al., 2011). Read mapping to SC2_gp020 would not be expected if the SC2 prophage is absent in the studied Las strain, but reads were mapped to this exclusive gene. Nonetheless, the presence of only SC2_gp020 from the late region from SC2 might indicate rearrangement among 3′ and 5′ of the SC2 prophage, as observed for the Las strain 9PA, and besides that, genes exclusive to the 5′ of SC2 are also present in Las Brazilian strains (Silva et al., 2019, 2021). The expression of the SC1 prophage late genes was outstanding but not all genes were expressed, indicating specific transcription inhibition in the psyllid, as observed for SC1_gp110 (holin) (Jain et al., 2017a) and adjacent genes.

Read mapping of Las genes indicates a high number of genes being expressed in the initial step of *D. citri* gut colonization. Such genes should be essential for Las to colonize insect organs, since higher read coverage was observed after longer feeding periods in Las-affected plants. The low percentage of non-expressed genes indicates that the reduced genome of Las is highly active in transcription. Transcriptome studies should help define targets to better understand the interaction of Liberibacter with its hosts and to design strategies to better cope with HLB in the near future.

## Data Availability Statement

The data presented in the study is available in the Sequence Read Archive under the accession PRJNA722422 and accession number for each library is provided in Supplementary Table 4.

## Author Contributions

FC and NW designed the experiments. JD prepared plants, insects, and samples. BM, FB, and JD processed and analyzed sequencing data. JD and NW interpreted the data and drafted the manuscript. All authors commented on the initial draft and approved the final version of this manuscript. FC, NW, and LP secured the funds.

## Conflict of Interest

The authors declare that the research was conducted in the absence of any commercial or financial relationships that could be construed as a potential conflict of interest.

## Footnotes

1 http://bioinformatics.psb.ugent.be/webtools/Venn/

## Supplementary Material

Supplementary Table 1 The list of exclusive coding sequences mapped only in Las III.

Supplementary Table 2 The list of genes without mapped reads.

Supplementary Table 3 The list of coding sequences for each Las library mapped against Las JXGC genome with FPKM values, average and log2FPKM used for analysis.

Supplementary Table 4 Accession number for each library.

## References

[B1] Abbà S. Galetto L. Carle P. Carrère S. Delledonne M. Foissac X. (2014). RNA-Seq profile of flavescence dorée phytoplasma in grapevine. *BMC Genom.* 15:1088. 10.1186/1471-2164-15-1088 25495145 PMC4299374

[B2] Ammar E. D. Ramos J. E. Hall D. G. Dawson W. O. Shatters R. G. Jr. (2016). Acquisition, Replication and Inoculation of *Candidatus* Liberibacter asiaticus following various acquisition periods on huanglongbing infected citrus by nymphs and adults of the asian citrus psyllid. *PLoS One* 11:e0159594. 10.1371/journal.pone.0159594.t006 PMC4956146 27441694

[B3] Ammar E. D. Robert G. Shatters R. G. Jr. Lynch C. Hall D. G. (2011a). Detection and relative titer of *Candidatus* Liberibacter asiaticus in the salivary glands and alimentary canal of *Diaphorina citri* (Hemiptera: Psyllidae) vector of citrus Huanglongbing disease. *Ann. Entomol. Soc. A.* 104 526–533. 10.1603/AN10134 33044624

[B4] Ammar E. D. Shatters R. G. Jr. Hall D. G. (2011b). Localization of *Candidatus* Liberibacter asiaticus, associated with citrus Huanglongbing disease, in its psyllid vector using fluorescence in situ hybridization. *J. Phytopathol.* 159 726–734. 10.1111/j.1439-0434.2011.01836.x

[B5] Andrade M. Wang N. (2019). The Tad Pilus apparatus of ‘*Candidatus* Liberibacter asiaticus’ and its regulation by VisNR. *Mol. Plant Microbe. Interact.* 32 1175–1187. 10.1094/MPMI-02-19-0052-R 30925227

[B6] Andrade M. O. Pang Z. Achor D. S. Wang H. Yao T. Singer B. H. (2019). The flagella of ‘*Candidatus* Liberibacter asiaticus’ and its movement in planta. *Mol. Plant Pathol.* 21 109–123. 10.1111/mpp.12884 31721403 PMC6913195

[B7] Andrews S. (2010). *FastQC: a Quality Control Tool for High Throughput Sequence Data*. Available online at: http://www.bioinformatics.babraham.ac.uk/projects/fastqc (accessed December 16, 2020).

[B8] Bassanezi R. B. Lopes S. A. Miranda M. P. Wulff N. A. Volpe H. X. L. Ayres A. J. (2020). Overview of citrus huanglongbing spread and management strategies in Brazil. *Trop Plant Pathol.* 45 251–264. 10.1007/s40858-020-00343-y

[B9] Bergamin Filho A. Inoue-Nagata A. K. Bassanezi R. B. Belasque J. Jr. Amorim L. Macedo M. A. (2016). The importance of primary inoculum and area-wide disease management to crop health and food security. *Food Sec.* 8 221–238. 10.1007/s12571-015-0544-8

[B10] Bolger A. M. Lohse M. Usadel B. (2014). Trimmomatic: a flexible trimmer for Illumina sequence data. *Bioinformatics* 30 2114–2120. 10.1093/bioinformatics/btu170 24695404 PMC4103590

[B11] Bové J. M. (2006). Huanglongbing: a destructive, newly emerging, century-old disease of citrus. *J. Plant Pathol.* 88 7–37. 10.4454/jpp.v88i1.828 32896216

[B12] Canale M. C. Tomaseto A. F. Haddad M. L. Coletta-Filho H. D. Lopes J. R. S. (2017). Latency and Persistence of ‘*Candidatus* Liberibacter asiaticus’ in Its Psyllid Vector, *Diaphorina citri (Hemiptera: Liviidae*). *Phytopathology* 107 264–272. 10.1094/PHYTO-02-16-0088-R 27841960

[B13] Capoor S. P. Rao D. G. Viswanath S. M. (1967). *Diaphorina citri*: a vector of the greening disease of citrus in India. *Indian J. Agricult. Sci.* 37 572–576.

[B14] Carmo-Souza M. Garcia R. B. Wulff N. A. Fereres A. Miranda M. P. (2020). Drench application of systemic insecticides disrupts probing behavior of *Diaphorina citri* (*Hemiptera: Liviidae*) and inoculation of *Candidatus* Liberibacter asiaticus. *Insects* 11:314. 10.3390/insects11050314 32429404 PMC7290861

[B15] Casjeans S. (2003). Prophages and bacterial genomics: what have we learned so far? *Mol. Microb.* 49 277–300. 10.1046/j.1365-2958.2003.03580.x 12886937

[B16] Cicero J. M. Fisher T. W. Brown J. K. (2016). Localization of ‘*Candidatus* Liberibacter solanacearum’ and evidence for surface appendages in potato psyllid vector. *Phytopathology* 106 142–154. 10.1094/PHYTO-04-15-0088-R 26551449

[B17] Cicero J. M. Fisher T. W. Qureshi J. A. Stansly P. A. Brown J. K. (2017). Colonization and intrusive invasion of potato psyllid by ‘*Candidatus* Liberibacter solanacearum’. *Phytopathology* 107 36–49. 10.1094/PHYTO-03-16-0149-R 27482628

[B18] Cifuentes-Arenas J. C. Goes A. Miranda M. P. Beattie G. A. C. Lopes S. A. (2018). Citrus flush shoot ontogeny modulates biotic potential of *Diaphorina citri*. *PLoS One* 13:e0190563. 10.1371/journal.pone.0190563 29304052 PMC5755881

[B19] Creecy J. P. Conway T. (2015). Quantitative bacterial transcriptomics with RNA-seq. *Curr. Opin. Microbiol.* 23 133–140. 10.1016/j.mib.2014.11.011 25483350 PMC4323862

[B20] Croucher N. J. Thomson N. R. (2010). Studing bacterial trancriptomes using RNA-seq. *Curr. Opin. Microbiol.* 13 619–624. 10.1016/j.mib.2010.09.009 20888288 PMC3025319

[B21] Cruz-Munoz M. Munoz-Beristain A. Petrone J. R. Robinson A. M. Triplett E. W. (2019). Growth parameters of *Liberibacter crescens* suggest ammonium and phosphate as essential molecules in the Liberibacter-plant host interface. *BMC Microbiol.* 19:222. 10.1186/s12866-019-1599-z 31606047 PMC6790036

[B22] Dossi F. C. A. Silva E. P. Cônsoli F. L. (2014). Population dynamics and growth rates of endosymbionts during *Diaphorina citri* (*Hemiptera*. *Liviidae*) ontogeny. *Microbiol. Ecol.* 68 881–889. 10.1007/s00248-014-0463-9 25037159

[B23] Duan Y. Zhou L. Hall D. G. Li W. Doddapaneni H. Lin H. (2009). Complete genome sequence of Citrus Huanglongbing bacterium, ‘*Candidatus* Liberibacter asiaticus’ obtained through metagenomics. *Mol. Plant Microbe. Interact.* 22 1011–20. 10.1094/MPMI-22-8-1011 19589076

[B24] Fleites L. Jain M. Zhang S. Gabriel D. W. (2014). “*Candidatus* Liberibacter asiaticus” prophage late genes may limit host range and culturability. *Appl. Environ. Microbiol.* 80 6023–6030. 25063651 10.1128/AEM.01958-14 PMC4178692

[B25] Fu S. Shao J. Zhou C. Hartung J. S. (2016). Transcriptome analysis of sweet orange trees infected with ‘*Candidatus* Liberibacter asiaticus’ and two strains of Citrus Tristeza Virus. *BMC Genomics* 17:349. 10.1186/s12864-016-2663-9 27169471 PMC4865098

[B26] Garnier M. Danel N. Bové J. M. (1984). *The Greening Organism is a Gran Negative Bacterium.* United States: University of California.

[B27] Ghanim M. Achor D. Ghosh S. Kontsedalov S. Lebedev G. Levy G. (2017). ‘*Candidatus* Liberibacter asiaticus’ accumulates inside endoplasmic reticulum associated vacuoles in the gut cells of *Diaphorina citri*. *Sci. Rep.* 7:16945. 10.1038/s41598-017-16095-w 29208900 PMC5717136

[B28] Ghosh P. K. Maiti T. K. (2016). Structure of extracellular polysaccharides (EPS) produced by Rhizobia and their functions in legume-bacteria symbiosis: a review. *Achiev. Life Sci.* 10 136–143. 10.1016/j.als.2016.11.003

[B29] Graninger M. Nidetzky B. Heinrichs D. E. Whitfield C. Messner P. (1999). Characterization of dTDP-4-dehydrorhamnose 3,5-epimerase and dTDP-4-dehydrorhamnose reductase, required for dTDP-L-rhamnose biosynthesis in *Salmonella enterica* serovar typhimurium LT2. *J. Biol. Chem.* 274 25069–25077. 10455186 10.1074/jbc.274.35.25069

[B30] Guidolin A. S. Cônsoli F. L. (2013). Molecular characterization of *Wolbachia* strains associated with the invasive asian citrus psyllid *Diaphorina citri* in Brazil. *Microb. Ecol.* 65 475–486. 10.1007/s00248-012-0150-7 23269454

[B31] Haas B. J. Chin M. Nusbaum C. Birren B. W. Livny J. (2012). How deep is deep enough for RNA-Seq profiling of bacterial transcriptomes? *BCM Genom.* 13:734. 10.1186/1471-2164-13-734 23270466 PMC3543199

[B32] Hu Y. Zhong X. Liu X. Lou B. Zhou C. Wang X. (2017). Comparative transcriptome analysis unveils the tolerance mechanisms of *Citrus hystrix* in response to ‘*Candidatus* Liberibacter asiaticus’ infection. *PLoS One* 12:e0189229. 10.1371/journal.pone.0189229 29232716 PMC5726760

[B33] Hung T. H. Hung S. C. Chen C. N. Hsu M. H. Su H. J. (2004). Detection by PCR of *Candidatus* Liberibacter asiaticus, the bacterium causing citrus Huanglongbing in vector psyllids: application to the study of vector-pathogen relationships. *Plant Pathol.* 53 96–102. 10.1111/j.1365-3059.2004.00948.x

[B34] Ibanez F. Levy J. Tamborindeguy C. (2014). Transcriptome analysis of “*Candidatus* Liberibacter solanacearum” in its Psyllid Vector, *Bactericera cockerelli*. *PLoS One* 9:e100955. 10.1371/journal.pone.0100955 24992557 PMC4081026

[B35] Inoue H. Ohnish I. J. Ito T. Tomimura K. Miyata S. Iwanami T. (2009). Enhanced proliferation and efficient transmission of *Candidatus* Liberibacter asiaticus by adult *Diaphorina citri* after acquisition feeding in the nymphal stage. *Ann. Appl. Biol.* 155 29–36. 10.1111/j.1744-7348.2009.00317.x

[B36] Jagoueix S. Bové J. M. Garnier M. (1994). The phloem-limited bacterium of greening disease of citrus is a member of the alpha subdivision of the *Proteobacteria*. *Int. J. Syst. Bacteriol.* 44 379–386. 10.1099/00207713-44-3-379 7520729

[B37] Jain M. Fleites L. A. Gabriel D. W. (2015). Prophage-encoded peroxidase in ‘*Candidatus* Liberibacter asiaticus’ is a secreted effector that suppresses plant defenses. *Mol. Plant Microbe. Interact.* 28 1330–1337. 10.1094/MPMI-07-15-0145-R 26313412

[B38] Jain M. Fleites L. A. Gabriel D. W. (2017a). A small *Wolbachia* protein directly represses phage lytic cycle genes in “*Candidatus* Liberibacter asiaticus” within psyllids. *mSphere* 2 e00171–17. 10.1128/mSphere.00227-17 28608866 PMC5463029

[B39] Jain M. Munoz-Bodnar A. Gabriel D. W. (2017b). Concomitant loss of the glyoxalase system and glycolysis makes the uncultured pathogen “*Candidatus* Liberibacter asiaticus” an energy scavenger. *Appl. Environ. Microbiol.* 83 e01670–17. 10.1128/AEM.01670-17 28939611 PMC5691416

[B40] Katoh H. Miyata S. Inoue H. Iwanami T. (2014). Unique features of a japanese ‘*Candidatus* Liberibacter asiaticus’ strain revealed by whole genome sequencing. *PLoS One* 9:e106109. 10.1371/journal.pone.0106109 25180586 PMC4152171

[B41] Kolora L. D. Powell C. M. Hunter W. Bextine B. Lauzon C. R. (2015). Internal extracellular bacteria of *Diaphorina citri* Kuwayama (*Hemiptera: Psyllidae*), the asian citrus psyllid. *Curr. Microb.* 70 710–715. 10.1007/s00284-015-0774-1 25645736

[B42] Konstantinidis K. T. Tiedje J. M. (2004). Trends between gene content and genome size in prokaryotic species with larger genomes. *Proc. Nat. Acad. Sci. U. S. A.* 101 3160–3165. 10.1073/pnas.0308653100 14973198 PMC365760

[B43] Kruse A. Fattah-Hosseini S. Saha S. Johnson R. Warwick E. R. Sturgeon K. (2017). Combining ‘omics and microscopy to visualize interactions between the asian citrus psyllid vector and the huanglongbing pathogen *Candidatus* Liberibacter asiaticus in the insect gut. *PLoS One* 12:e0179531. 10.1371/journal.pone.0179531 28632769 PMC5478155

[B44] Kumar N. Lin M. Zhao X. Ott S. Santana-Cruz I. Daugherty S. (2015). Efficient enrichment of bacterial mRNA from host-bacteria total RNA samples. *Sci. Rep.* 6:34850. 10.1038/srep34850 27713560 PMC5054355

[B45] Lee J. A. Halbert S. E. Dawson W. O. Robertson C. J. Keesling J. E. Singer B. H. (2015). Asymptomatic spread of huanglongbing and implications for disease control. *Proc. Nat. Acad. Sci. U. S. A.* 112 7605–7610. 10.1073/pnas.1508253112 26034273 PMC4475945

[B46] Leonard M. T. Fagen J. R. Davis-Richardson A. G. Davis M. J. Triplett E. W. (2012). Complete genome sequence of *Liberibacter crescens* BT-1. *Stand. Genomic. Sci.* 7 271–283. 10.4056/sigs.3326772 23408754 PMC3569387

[B47] Li J. Pang Z. Trivedi P. Zhou X. Ying X. Jia H. (2017). “*Candidatus* Liberibacter asiaticus” encodes a functional salicylic acid (SA) hydroxylase that degrades SA to suppress plant defence. *Mol. Plant Microbe. Interact.* 8 620–630. 10.1094/MPMI-12-16-0257-R 28488467

[B48] Li T. Zhang L. Deng Y. Deng X. Zheng Z. (2021). Establishment of a *Cuscuta campestris*-mediated enrichment system for genomic and transcriptomic analysis of ‘*Candidatus* Liberibacter asiaticus’. *Microbial. Biotec.* 2021 737–751. 10.1111/1751-7915.13773 33655703 PMC7936317

[B49] Li W. B. Hartung J. S. Levy L. (2006). Quantitative real-time PCR for detection and identification of *Candidatus* Liberibacter species associated with citrus Huanglongbing. *J. Microbiol. Methods* 66 104–115. 10.1016/j.mimet.2005.10.018 16414133

[B50] Liefting L. W. Perez-Egusquiza Z. C. Clover G. R. G. Anderson J. A. D. (2008). A new ‘*Candidatus* Liberibacter’ species in *Solanum tuberosum* in New Zealand. *Plant Dis.* 92:1474. 10.1094/PDIS-92-10-1474A 30769561

[B51] Lin H. Han C. S. Liu B. Lou B. Bai X. Deng C. (2013). Complete genome sequence of a chinese strain of ‘*Candidatus* Liberibacter asiaticus’. *Genome Announc.* 1 e00184–13. 10.1128/genomeA.00184-13 23640196 PMC3642251

[B52] Lin H. Lou B. Glynn J. M. Doddapaneni H. Civerolo E. L. Chen C. (2011). The complete genome sequence of ‘*Candidatus* Liberibacter solanacearum’, the bacterium associated with potato zebra chip disease. *PLoS One* 6:e19135. 10.1371/journal.pone.0019135 21552483 PMC3084294

[B53] Lin H. Pietersen G. Han C. Read D. A. Lou B. Gupta G. (2015). Complete genome sequence of “*Candidatus* Liberibacter africanus,” a bacterium associated with citrus Huanglongbing. *Genome Announc.* 3 e00733–15. 10.1128/genomeA.00733-15 26184931 PMC4505119

[B54] Liu X. Zheng Y. Wang-Pruski G. Gan Y. Zhang B. Hu Q. (2019). Transcriptome profiling of periwinkle infected with Huanglongbing (‘*Candidatus* Liberibacter asiaticus’). *Eur. J. Plant Pathol.* 153 891–906. 10.1007/s10658-018-01607-9

[B55] Liu X.-Q. Jiang H.-B. Liu T.-Y. Yang L. Fan J.-Y. Xiong Y. (2020). A Transcriptomic and Proteomic analysis of the *Diaphorina citri* salivary glands reveals genes responding to *Candidatus* Liberibacter asiaticus. *Front. Physiol.* 11:582505. 10.3389/fphys.2020.582505 33101062 PMC7546269

[B56] Lopes S. A. Cifuentes-Arenas J. C. (2021). A protocol for successful transmission of ‘*Candidatus* Liberibacter asiaticus’ from citrus to citrus using *Diaphorina citri*. *Phytopathology* 10.1094/PHYTO-02-21-0076-R [Epub ahead of print]. 33938771

[B57] Manjunath K. L. Halbert S. E. Ramadugu C. Webb S. Lee R. F. (2008). Detection of *Candidatus* Liberibacter asiaticus” in *Diaphorina citri* and its importance in the management of Citrus Huanglongbing in Florida. *Phytopathology* 98 387–396. 10.1094/PHYTO-98-4-0387 18944186

[B58] Martinelli A. F. Uratsu S. L. Albrecht U. Reagan R. L. Phu M. P. Britton M. (2012). Transcriptome profiling of citrus fruit response to huanglongbing disease. *PLoS One* 7:e38039. 10.1371/journal.pone.0038039 22675433 PMC3364978

[B59] Mira A. Ochman H. Moran N. A. (2001). Deletional bias and the evolution of bacterial genomes. *Trends Genet.* 17 589–596. 10.1016/S0168-9525(01)02447-7 11585665

[B60] Miranda M. P. Ayres A. J. (2020). “Asian citrus psyllid management in São Paulo, Brazil” in *Asian Citrus Psyllid: biology, Ecology and Management of Huanglongbing Vector.* eds Qureshi J. Stansly P.A. (Wallingford: CABI).

[B61] Mohamed A. R. Chan C. X. Ragan M. A. Zhang J. Cooke I. Ball E. E. (2020). Comparative transcriptomic analyses of *Chromera* and Symbiodiniaceae. *Environ. Microbiol. Rep.* 12 435–443. 10.1111/1758-2229.12859 32452166

[B62] Munyaneza J. E. Sengoda V. G. Crosslin J. M. Garzón-Tiznado J. A. Cardenas-Valenzuela O. G. (2009). First report of “*Candidatus* Liberibacter solanacearum” in tomato plants in Mexico. *Plant Dis.* 93:552. 10.1094/PDIS-93-10-1076A 30754366

[B63] Nakabachi A. Nikoh N. Oshima K. Inoue H. Ohkuma M. Hongoh Y. (2013a). Horizontal gene acquisition of Liberibacter plant pathogens from a bacteriome-confined endosymbiont of their psyllid vector. *PloS One* 8:e82612. 10.1371/journal.pone.0082612 24349319 PMC3857777

[B64] Nakabachi A. Ueoka R. Oshima K. Teta R. Mangoni A. Gurgui M. (2013b). Defensive bacteriome symbiont with a drastically reduced genome. *Curr. Biol.* 23 1478–1484. 10.1016/j.cub.2013.06.027 23850282

[B65] Naranjo E. Merfa M. V. Ferreira V. Jain M. Davis M. J. Bahar O. (2019). *Liberibacter crescens* biofilm formation *in vitro*: establishment of a model system for pathogenic ‘*Candidatus* Liberibacter spp.’. *Sci. Rep.* 9:5150. 10.1038/s41598-019-41495-5 30914689 PMC6435755

[B66] Pelz-Stelinski K. S. Brlansky R. H. Ebert T. A. Rogers M. E. (2010). Transmission parameters for *Candidatus* Liberibacter asiaticus by asian citrus psyllid (*Hemiptera: Psyllidae*). *J. Econ. Entomol.* 103 1531–41. 10.1603/EC10123 21061950

[B67] Porankiewicz J. Wang J. Clarke A. K. (1999). New insights into the ATP-dependent Clp protease: *escherichia coli* and beyond. *Mol. Microb.* 32 449–458. 10.1046/j.1365-2958.1999.01357.x 10320569

[B68] Prasad S. Xu J. Zhang Y. Wang N. (2016). SEC-translocon dependent extracytoplasmic proteins of *Candidatus* Liberibacter asiaticus. *Front. Microbiol.* 7:1989. 10.3389/fmicb.2016.01989 28066334 PMC5167687

[B69] Sällsträm B. Andersson G. E. (2005). Genome reduction in the α-*Proteobacteria*. *Curr. Opin. Microbiol.* 8 579–585. 10.1016/j.mib.2005.08.002 16099701

[B70] Selvaraj V. Maheshwari Y. Hareji S. Chen J. McCollum T. G. Yokomi R. (2018). Development of a duplex droplet digital PCR assay for absolute quantitative detection of ‘*Candidatus* Liberibacter asiaticus’. *PLoS One* 13:e0197184. 10.1371/journal.pone.0197184 29772016 PMC5957411

[B71] Silva P. A. Fassini C. G. Sampaio L. S. Dequigiovanni G. Zucchi M. A. Wulff N. A. (2019). Genetic diversity of *Candidatus* Liberibacter asiaticus revealed by short tandem repeats and prophages typing indicates population homogeneity in Brazil. *Phytophathology* 109 960–971. 10.1094/PHYTO-08-18-0295-R 30694114

[B72] Silva P. A. Huang J. Wulff N. A. Zheng Z. Krugner R. Chen J. (2021). Genome sequence resource of ‘*Candidatus* Liberibacter asiaticus’ strain 9PA from Brazil. *Plant Dis.* 105 199–201. 10.1094/PDIS-05-20-1018-A 32697180

[B73] Spero M. A. Aylward F. O. Currie C. R. Donohue T. J. (2015). Phylogenomic analysis and predicted physiological role of the proton-translocating NADH:quinone oxidoreductase (Complex I) across bacteria. *mBio* 6 389–315. 10.1128/mBio.00389-15 25873378 PMC4453560

[B74] Subandiyah S. Nikoh N. Tsuyumu S. Somowiyarjo S. Fukatsu T. (2000). Complex endosymbiotic microbiota of the citrus psyllid *Diaphorina citri* (*Homoptera: Psylloidea*). *Zool. Sci.* 17 983–989. 10.2108/zsj.17.983

[B75] Tatusov R. M. Gasperim M. Y. Natale D. A. Koonin E. V. (2000). The COG database: a tool for genome-scale analysis of protein functions and evolution. *Nucl. Acid Res.* 28:1. 10.1093/nar/28.1.33 10592175 PMC102395

[B76] Teixeira D. C. Colette S. Carole C. Martins E. C. Wulff N. A. Jagoueix S. E. (2008). Distribution and quantification of *Candidatus* Liberibacter americanus, agent of Huanglongbing disease of citrus in São Paulo State, Brasil, in leaves of an affected sweet orange tree as determined by PCR. *Mol. Cell. Probes* 22 139–150. 10.1016/j.mcp.2007.12.006 18400468

[B77] Thapa S. P. De Francesco A. Trinh J. Gurung F. B. Pang Z. Vidalakis G. (2020). Genome-wide analyses of Liberibacter species provides insights into evolution, phylogenetic relationships, and virulence factors. *Mol. Plant Pathol.* 21 716–731. 10.1111/mpp.12925 32108417 PMC7170780

[B78] Tomimura K. Miyata S. Furuya N. Kubota K. Okud A. M. Subandiyah S. (2009). Evaluation of genetic diversity among ‘*Candidatus* Liberibacter asiaticus’ isolates collected in Southeast Asia. *Phytopathogy* 99 1062–9. 10.1094/PHYTO-99-9-1062 19671008

[B79] Ukuda-Hosokawa R. Sodayama Y. Kishaba M. Kuriwada T. Anbutsu H. Fukatsu T. (2015). Infection density dynamics of the citrus greening bacterium “*Candidatus* Liberibacter asiaticus” in field populations of the psyllid *Diaphorina citri* and its relevance to the efficiency of pathogen transmission to citrus plants. *Appl. Environ. Microbiol.* 81 3728–3736. 10.1128/AEM.00707-15 25819961 PMC4421049

[B80] Wairuri C. K. Van der Waals J. E. Van Schalkwyk A. Theron J. (2012). *Ralstonia solanacearum* needs Flp Pili for virulence on potato. *Mol. Plant Microbe Interact.* 25 546–556. 10.1094/MPMI-06-11-0166 22168446

[B81] Wang Y. Kondo T. He Y. Zhou Z. Lu J. (2021). Genome sequence resource of ‘*Candidatus* Liberibacter asiaticus’ from *Diaphorina citr*i Kuwayama (*Hemiptera: Liviidae*) in Colombia. *Plant Dis.* 105 193–195. 10.1094/PDIS-06-20-1249-A 32729808

[B82] Wulff N. A. Daniel B. Sassi R. S. Moreira A. S. Bassanezi R. B. Sala I. (2020). Incidence of *Diaphorina citri* carrying *Candidatus* Liberibacter asiaticus in Brazil’s citrus belt. *Insects* 11:672. 10.3390/insects11100672 33022967 PMC7650542

[B83] Wulff N. A. Zhang S. Setubal J. Almeida N. F. Martins E. C. Harakava R. (2014). The complete genome sequence of *Candidatus* Liberibacter americanus, associated with citrus huanglongbing. *Mol. Plant Microbe Interact.* 27 163–176. 10.1094/MPMI-09-13-0292-R 24200077

[B84] Yan Q. Sreedharan A. Wei S. Wang J. Pelz-Stelinski K. Folimonova S. (2013). Global gene expression changes in *Candidatus* Liberibacter asiaticus during the transmission in distinct host between plant and insects. *Mol. Plant Pathol.* 14 391–404. 10.1111/mpp.12015 23336388 PMC6638839

[B85] Zhang S. Flores-Cruz Z. Zhou L. Kang B. H. Fleites L. A. Gooch M. D. (2011). Ca. Liberibacter asiaticus’ carries an excision plasmid prophage and a chromosomally integrated prophage that becomes lytic in plant infections. *Mol. Plant Microbe Interact.* 24 458–468. 10.1094/MPMI-11-10-0256 21190436

[B86] Zheng Y. Huang H. Huang Z. Deng X. Zheng Z. Xu M. (2021). Prophage region and short tandem repeats of “*Candidatus* Liberibacter asiaticus” reveal significant population structure in China. *Plant Pathol.* 70 959–969 10.1111/ppa.13332

[B87] Zheng Z. Bao M. Wu F. Van Horn C. Chen J. Deng X. (2018). A type 3 prophage of ‘*Candidatus* Liberibacter asiaticus’ carring a restricition-modification system. *Phytopathology* 108 454–461. 10.1094/PHYTO-08-17-0282-R 29192841

[B88] Zuñiga C. Peacock B. Liang B. McCollum G. Irigoyen S. C. Tec-Campos D. (2020). Linking metabolic phenotypes to pathogenic traits among “*Candidatus* Liberibacter asiaticus” and its hosts. *Syst. Biol. Appl.* 6:24. 10.1038/s41540-020-00142-w 32753656 PMC7403731

